# Surgical excision of an abdominal wall granular cell tumour with Permacol^® ^mesh reconstruction: a case report

**DOI:** 10.1186/1477-7800-5-4

**Published:** 2008-02-25

**Authors:** Aisha Chaudhry, Ewen A Griffiths, Nigam Shah, Srinivasan Ravi

**Affiliations:** 1 Department of General Surgery, Blackpool Victoria Hospital, Blackpool Fylde and Wyre NHS Trust, Blackpool, FY2 8NR, UK; 2 Department of Histopathology, Blackpool Victoria Hospital, Blackpool Fylde and Wyre NHS Trust, Blackpool, FY2 8NR, UK

## Abstract

**Introduction:**

Granular cell tumours of the abdominal wall are extremely rare: less than 10 have been reported in the worldwide medical literature. We report this interesting case, review the relevant literature on this tumour and discuss surgical abdominal wall reconstruction options.

**Case presentation:**

A 70 year old lady presented with a left abdominal mass. This was thought to be a soft tissue sarcoma on CT imaging prior to surgical excision. En-bloc surgical resection was performed. Surgical reconstruction of the abdominal wall defect was performed using Permacol^® ^mesh. Histopathological examination of the surgical specimen showed it to be a granular cell tumour.

**Conclusion:**

Although rare, granular cell tumours can present as an abdominal wall mass. It is important that clinicians are aware of their existence. The closure of large defects, after surgical resection of abdominal wall tumours, is a surgical challenge. We used a new biosynthetic procine mesh (Permacol^®^) which appeared to work well in this situation.

## Introduction

Granular cell tumours (GCT) are rare neural tumours characterized by large granular appearing eosinophilic cells. GCTs were initially considered to be of striated muscle origin by Abrikossoff who described a tumour arising from the tongue in 1926 [1]. Older terms for this tumour type include granular cell myoblastoma, granular cell neuroma, granular cell neurofibroma and granular cell schwannoma. However, newer investigational techniques such as electron microscopy and immunnohistochemistry have proven that GCTs are most likely derived from Schwannoma cells of the peripheral nerve fibres [2,3]. Most GCTs are benign, but rare malignant types have been reported. The tongue is the single most common anatomical site, but GCTs can be found in virtually any body site, including skin, subcutaneous tissue, breast, rectum, oesophagus and vulva [4]. Previously only seven cases of abdominal wall GCTs have been reported in the medical literature [5].

We describe a new case of a GCT arising from the abdominal wall muscles in a 70 year old lady. We briefly review the medical literature on this tumour and discuss the surgical abdominal wall reconstruction options pertinent to this case.

## Case presentation

A 70 year old lady was referred urgently to the colorectal clinic with a palpable abdominal mass. She presented to her General Practitioner (GP) with left sided abdominal pain, diarrhoea and weight loss. Her GP found a suspicious left sided abdominal mass on examination and referred her urgently under the two week colorectal cancer rule. Her past medical history included hypertension, chronic obstructive airways disease and appendicectomy. She also had a previous total abdominal hysterectomy and bilateral salpingo-oopherectomy for large uterine fibroids.

**Figure 1:**
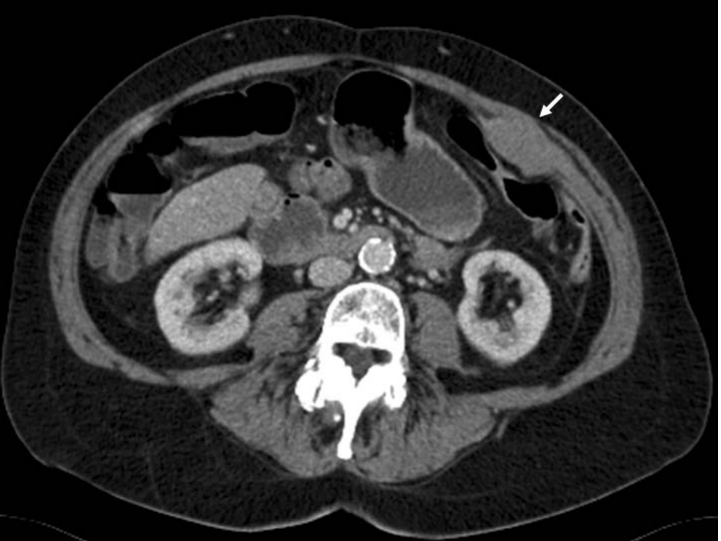
Computer Tomography showed an abdominal wall tumour (arrowed) arising from the left anterior abdominal wall muscles, in particular the internal oblique and transversus abdominis.

**Figure 2:**
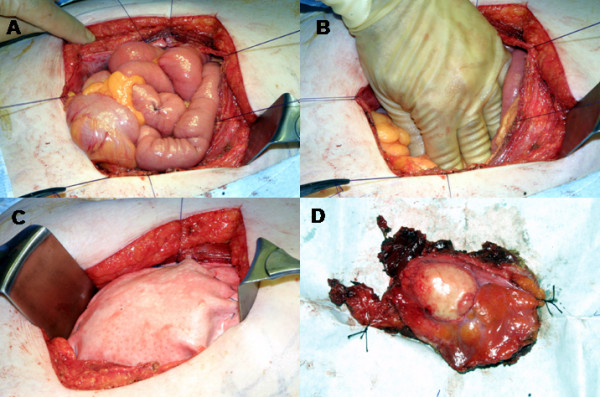
**This figure documents the surgical resection.** (A and B) shows the abdomen after the en-bloc resection of the abdominal wall tumour. Stay sutures are shown on the edges of the large surgical defect. (C) The Permacol^® ^mesh has been sutured on to the inner layer of the abdominal wall in direct contact with the bowel. (D) The peritoneal surface of the excised surgical specimen is shown.

On examination in the outpatient clinic a 10 × 7 cm firm, fixed lump was found in the left iliac fossa area of the abdomen. Urgent colonoscopic examination revealed mild sigmoid diverticular disease with no evidence of colonic malignancy. Computer Tomography was preformed (Figure 1) and showed the mass to be arising from the anterior abdominal wall muscles, in particular the internal oblique and transversus abdominis. There was no evidence of distant metastatic disease to the liver or lungs. The clinical suspicion was of a malignant abdominal wall sarcoma. Fine needle aspiration or percutaneous biopsy was not performed. En-bloc surgical resection of the tumour was performed via a left flank incision (Figure 2). At surgical resection the tumour mass involved the internal oblique, transversus abdominis and there was a small area of peritoneal ulceration. No distant disease was found at surgery. The tumour was excised en-bloc with a surrounding margin of healthy tissue (Figure 2). Part of the external oblique aponeurosis was preserved to allow adequate closure. The large abdominal wall defect was closed using a sheet of Permacol^® ^mesh (Tissue Science Laboratories plc, Hampshire, England). The Permacol^® ^mesh was sutured to the posterior leaf of the rectus sheath medially and the internal oblique laterally using a slow absorbing polydioxanone suture. The remaining external oblique muscle was closed over the mesh and the subcutaneous tissue and skin were closed in a standard fashion.

**Figure 3:**
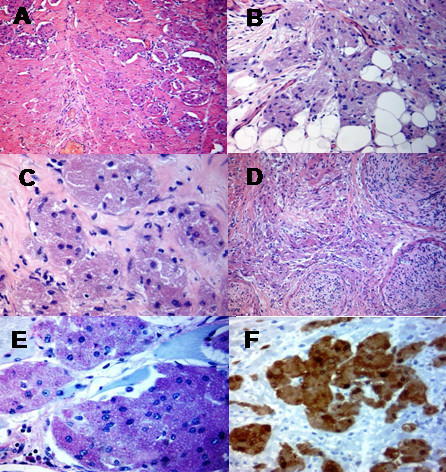
**The microscopic features of the granular cell tumour were: (A) Round polygonal granular cells observed in nests, divided by fibrous septa; (B) The tumour had an infiltrating margin; (C) The nuclei were small and dark**. The cytoplasm was eosinophilic fine to coarsely granular; (D) The granular cell tumour arose from the nerve bundles; (E) Histochemical stain showed the granules represent phagolysosomes which were strongly Periodic Acid Schiff (PAS) positive and diastase-resistant; (F) Immunohistochemsitry was strongly positive for S100.

The surgical specimen measured 11 × 7 × 4 cm. In the centre of the specimen, there was a 4 cm whitish solid tumour. The tumour consisted of relatively large cells with granular eosinophilic cytoplasm and small pleomorphic nuclei with occasional nucleoli (Figure 3). No conspicuous mitotic activity was noted. The tumour was completely resected within generous margins of normal tissue. The tumour cells showed strong positive reaction with S100 (Figure 3) and were negative with GFAP. The appearances were therefore consistent with a GCT.

This patient has been reviewed in the outpatient clinic and is currently alive and well five months after surgery.

## Discussion

GCTs typically present as a small, poorly circumscribed, painless, non-ulcerating nodule. The tumours can arise from virtually any anatomical site; the most common sites are the head and neck (especially tongue), the chest wall and the arm. They are mainly located in the dermis or the subcutaneous tissue (66% of cases) and less frequently submucosa, smooth muscle or striated muscle. They also can arise in the internal organs: larynx, bronchus, stomach, anogenital region and bile ducts[4]. They are mainly solitary, although they can be multiple (10–15%). They can occur at any age, but are most common in the fourth, fifth and sixth decades of life and are rare in children [6]. Females tend to be affected twice as frequently as males.

The histopathological differential diagnosis can include rhabdomyoma (contains cross-striations and glycogen); hibernoma and fibroxanthoma (contain lipid droplets); and reactive changes with surgical trauma (associated with inflammatory elements and areas of necrosis). A common site for a rhabdomyoma is the heart, which is helpful in the differential diagnosis. GCTs can also be mistaken for squamous cell carcinoma due to marked acanthosis and pseudoepitheliomatous hyperplasia. Immunohistochemistry is essential for the correct diagnosis [7]. Most GCTs stain positive for S100, neuron-specific enolase, NK1-C3 and CD68. They stain negative for epithelial, endothelial and smooth muscle markers.

Patients with completely resected benign lesions are usually cured. Excluding malignant tumours, recurrence is rare (it is more common if complete surgical excision is not achieved). Therefore the prognosis for these tumours appears to be favourable. There is no role for adjuvant therapy with radiotherapy or chemotherapy.

Malignant GCTs are encountered in only 2% of cases [8]. Only around 80 cases have been reported worldwide. They are characterised by necrosis, spindling, vesicular nuclei with prominent nucleoli, increased mitotic activity (> 2 mitoses/10 high power fields), a high nuclear to cytoplasmic ratio and pleomorphism. More than 3 of these features are required for a diagnosis of malignancy. Less than 3 of these features would classify the tumour as an atypical GCT. The prognosis of malignant GCT is poor with risk of local recurrence and distant metastases to lung, liver and bone. Interestingly, four of the reported seven GCTs of the abdominal wall have had malignant features. Our case showed the typical histopathological features of a GCT with no suspicious features of malignancy. However, given the high percentage of malignant lesions reported to be arising from the abdominal wall careful histopathological examination of this anatomical site is recommended.

Extensive abdominal wall defects like the one encountered here are a surgical challenge [9]. The options for surgical closure included: primary closure, the use of prosthetic mesh material and advanced plastic surgical techniques [10]. Primary closure has a high risk of future incisional hernia formation. Plastic surgical techniques (such as pedicled or free myocutaneous flaps from various sites including the rectus abdominis, latissumus dorsi or gracilis muscles) require advanced plastic surgical training and expertise.

The ideal material for the repair of abdominal wall defects would provide strength, flexibility, host tissue incorporation, and infection resistance. There is a wide variety of mesh material available for abdominal wall reconstruction [11]. Complications associated with the use of permanent synthetic biomaterials (such as prolene mesh) include foreign body reaction, small bowel obstruction, intestinal fistulation, erosion of the mesh material into adjacent abdominal viscera, and mesh shrinkage or migration [12]. These risks are particularly high when permanent mesh is placed in direct contact with the bowel and abdominal viscera. However, the newer biosynthetic material meshes, such as Permacol (manufactured from porcine collagen) or Alloderm (manufactured from human cadaveric collagen), do not appear to be at risk of this problem [13]. Permacol mesh is manufactured from acellular porcine collagen and is non-allergenic [9]. Its principal benefits include less adhesion formation, good incorporation into host tissue and resistance to infection. It does not elicit a foreign body response and is readily colonised by host tissue cells and blood vessels thus making it resistant to infection. It is soft and flexible yet has high tensile strength and is available in various sizes, making it ideal for closing abdominal wall defects. It has potential uses in complex incisional hernia repair, para-stomal hernia repair, abdominal wall reconstruction and cosmetic surgery.

## Conclusion

Although rare, GCTs can present as an abdominal wall mass. It is important that clinicians are aware of their existence. The closure of large defects, after surgical resection of abdominal wall tumours, is a surgical challenge. We used a new biosynthetic porcine mesh (Permacol^®^) to close the large abdominal wall defect and this appeared to be ideal for this particular situation.

## Competing interests

The author(s) declare that they have no competing interests.

## Authors' contributions

AC wrote the draft of the manuscript; EG assisted in the surgical procedure, performed the literature research and revised the manuscript; NS examined the surgical specimen, wrote the pathological report and helped with revising the manuscript; SR performed the surgical procedure and revised the manuscript for intellectual content.

## Consent

Written informed consent was obtained from the patient for publication of this Case report and accompanying images. A copy of the written consent is available for review by the Editor-in-Chief of this journal.
